# Biotechnological Potential of Cold Adapted *Pseudoalteromonas* spp. Isolated from ‘Deep Sea’ Sponges

**DOI:** 10.3390/md15060184

**Published:** 2017-06-19

**Authors:** Erik Borchert, Stephen Knobloch, Emilie Dwyer, Sinéad Flynn, Stephen A. Jackson, Ragnar Jóhannsson, Viggó T. Marteinsson, Fergal O’Gara, Alan D. W. Dobson

**Affiliations:** 1 School of Microbiology, University College Cork, National University of Ireland, Cork T12 YN60, Ireland; erikborchert1987@gmail.com (E.B.); 113431842@umail.ucc.ie (E.D.); 113397541@umail.ucc.ie (S.F.); stevejackson71@hotmail.com (S.A.J.); f.ogara@ucc.ie (F.O.); 2 Department of Research and Innovation, Matís ohf., Reykjavik 113, Iceland; stephen@matis.is (S.K.); ragnar@matis.is (R.J.); viggo@matis.is (V.T.M.); 3 Biomerit Research Centre, University College Cork, National University of Ireland, Cork T12 YN60, Ireland; 4 School of Biomedical Sciences, Curtin Health Innovation Research Institute, Curtin University, Perth 6102, WA, Australia

**Keywords:** *Pseudoalteromonas*, enzymes, genomics, pan-genome

## Abstract

The marine genus *Pseudoalteromonas* is known for its versatile biotechnological potential with respect to the production of antimicrobials and enzymes of industrial interest. We have sequenced the genomes of three *Pseudoalteromonas* sp. strains isolated from different deep sea sponges on the Illumina MiSeq platform. The isolates have been screened for various industrially important enzymes and comparative genomics has been applied to investigate potential relationships between the isolates and their host organisms, while comparing them to free-living *Pseudoalteromonas* spp. from shallow and deep sea environments. The genomes of the sponge associated *Pseudoalteromonas* strains contained much lower levels of potential eukaryotic-like proteins which are known to be enriched in symbiotic sponge associated microorganisms, than might be expected for true sponge symbionts. While all the *Pseudoalteromonas* shared a large distinct subset of genes, nonetheless the number of unique and accessory genes is quite large and defines the pan-genome as open. Enzymatic screens indicate that a vast array of enzyme activities is expressed by the isolates, including β-galactosidase, β-glucosidase, and protease activities. A β-glucosidase gene from one of the *Pseudoalteromonas* isolates, strain EB27 was heterologously expressed in *Escherichia coli* and, following biochemical characterization, the recombinant enzyme was found to be cold-adapted, thermolabile, halotolerant, and alkaline active.

## 1. Introduction

The genus *Pseudoalteromonas* are a subgroup of Gram-negative Gammaproteobacteria with common features, including a requirement for Na^+^ ions, motility, and aerobic and chemoheterotrophic metabolism. The genus was first described by Gauthier and co-workers and separated from the genus *Alteromonas* [1]. The genus can be divided into either pigmented or non-pigmented species, with members of the genus being known to possess the ability to produce a wide array of bioactive compounds. The pigmented species, in particular, are known to produce a range of antimicrobial and antifouling compounds which display activity against a broad spectrum of organisms and have, as a result, been widely investigated in the past [2,3,4,5]. While the non-pigmented species are typically not antimicrobial producers, they are, however, versatile producers of an array of different extracellular enzymes that are of potential biotechnological interest [6,7,8,9,10]. *Pseudoalteromonas* are one of the most frequently isolated bacteria from marine environments [11] and are routinely found in association with various eukaryotic hosts in these environments such as tunicates [12], algae [13], sponges [14], mussels [15], pufferfish [16], as well as algae and marine plants [17,18]. They have also been isolated as free living in seawater [19], sea ice [20], and marine sediment [21].

The deep oceans as an ecosystem are of growing interest to the scientific community. While the mean depth of the oceans is 3800 m, about 50% is deeper than 3000 m. With only 5% of the ‘deep sea’ having been explored to date, it is clear that the biotechnological potential of this unique ecosystem has yet to be fully exploited [22,23,24]. We have previously reported on the microbial biodiversity of deep sea sponges sampled at depths of between 760 and 2900 m below sea level, indicating that the microbial community structures of these sponges may represent an untapped source of potential microbial biodiversity [25,26]. Bacterial and fungal communities from deep sea sediments also continue to receive attention, not only from an ecological standpoint [27], but also due to the ability of microorganisms isolated from this ecosystem to produce novel bioactive molecules [28,29] and enzymes of biotechnological importance [30,31]. Cold-active enzymes are of particular interest as they possess a range of structural features that promote flexibility at the active site, low substrate affinity, and high specific activity at low temperatures. These characteristics are important in industrial biocatalysis, not only from an energy savings standpoint, but also due to the fact that performing reactions at low temperatures prevents undesirable chemical side reactions which can occur at higher temperatures; while also allowing rapid thermal inactivation of these enzymes, due to their thermolabile properties [32,33].

*Pseudoalteromonas* strains have previously been reported to produce a number of cold adapted enzymes including DNA ligase [34,35], pectate lyase [36], β-galactosidase [6], subtilase [10], and agarase [9], with enzyme production in *Pseudoalteromonas haloplanktis* TAC125 and other *Pseudoalteromonas* strains namely sp. ANT506, sp. ANT178, sp. KMM701, and sp. CF6-2 in particular being studied in more detail [31,33]. The cold adaptation properties of *Pseudoalteromonas haloplanktis* TAC125 have been described with respect to its thioredoxin system [37,38] and the involvement of a single highly-active iron superoxide dismutase as a reactive oxygen species defense mechanism [39,40]. With this in mind, this study focused on the isolation and comparative genomics of three non-pigmented *Pseudoalteromonas* spp. isolated at different depths from both marine sponges and sediment in an effort to assess their biotechnological potential.

The genomes share a large pan-genome and have a considerable number of unique gene clusters, but only a small number of genes are associated with potential host interaction in all the investigated genomes, irrespective of whether or not they have been isolated from sponges, from deep sea sediment, or from ocean water. Furthermore, *Pseudoalteromonas* strains EB27, SK18, and SK20, isolated from deep sea sponges do not share a large number of genes that could be attributed to a symbiotic lifestyle. While the strains displayed cold-adapted growth characteristics, they are unlikely to be true psychrophiles. The three strains did however display a number of interesting enzyme activities including β-glucosidase, protease, and β-galactosidase activities and a heterologously expressed β-glucosidase from strain EB27 displayed properties that are favorable to industrial applications, such as alkaline pH optimum, halotolerance, and cold-adaptation.

## 2. Results

### 2.1. Enzymatic Activity Profile

The three *Pseudoalteromonas* spp. isolates displayed a range of different enzyme activity profiles (Table 1) in plate based screening assays. The *Poecillastra compressa* isolate EB27 (retrieved from a depth of 1480 m) displayed the greatest range of different activities, with the most prominent being β-glucosidase, protease, and cellulase activity. SK20 (*Inflatella pellicula*, 2900 m) exhibited strong β-galactosidase activity and SK18 (*Sericolophus hawaiicus*, 2129 m) displayed high levels of protease activity. All of the isolates displayed some lipolytic activity.

Semi-quantitative assays investigating protease (SK18, EB27) and β-galactosidase (SK20, EB27) activity were also performed (Supplementary Figure S1); however, these activities do not appear to be cold adapted.

To assess the general temperature–growth profile of the three isolates we performed growth experiments at different temperatures (4 °C, 23 °C, 28 °C, and 37 °C) and calculated the growth rate and doubling time (Table 2).

We decided to sequence the genomes of these three *Pseudoalteromonas* strains in an attempt to gain a better understanding of their biotechnological potential based on these preliminary extracellular enzyme profiles, together with the fact that other *Pseudoalteromonas* strains such as *Pseudoalteromonas haloplanktis* strains TAC125, TAE79, Sp22b, and AS-11 have all been shown to produce a large number of biotechnologically important biocatalysts [41]. In addition, representatives of the genus *Pseudoalteromonas* have also been shown to produce a broad array of bioactive molecules such as antibiotics, antitumor agents, and toxins/antitoxins [11,42,43,44,45].

### 2.2. Genome Sequencing and Assembly

The three *Pseudoalteromonas* genomes were sequenced on the MiSeq platform and the coverage obtained ranged from 196× to 230×. The number of identified coding DNA sequences (CDS) ranged from 3582 to 4012, with EB27 having the largest genome of 4.56 Mb and 4012 CDS and SK18 the smallest genome with 3.98 Mb and 3582 CDS (Table 3). The sequencing results fall within the size range of known *Pseudoalteromonas* spp. genomes (*Pseudoalteromonas haloplanktis* TAC125 3.85 Mb [46] to *Pseudoalteromonas atlantica* T6c 5.1 Mb [47,48]), and do not appear to display any unusual patterns which may relate to their host sponge origin, like genome size or differing GC content. Nonetheless, the genome of EB27 is approximately 10% larger than the other compared genomes.

### 2.3. Genome Comparison

**Figure 1:**
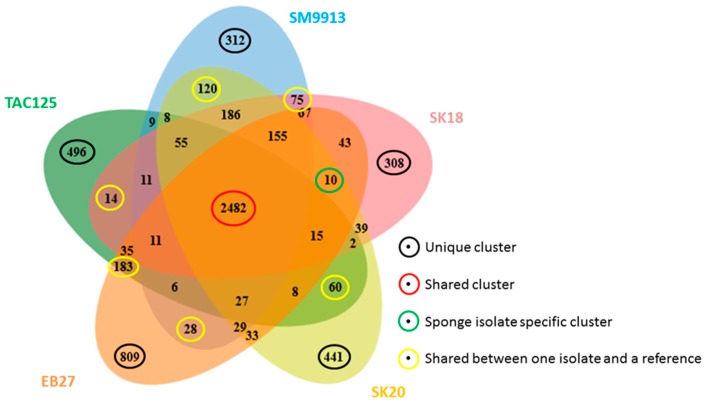
Whole genome comparison of translated non-redundant protein clusters from all three isolates and the two reference genomes (generated with [52]). Green coloured is *Pseudoalteromonas haloplanktis* TAC125, blue coloured *Pseudoalteromonas* sp. SM9913, light red coloured is SK18, yellow coloured is SK20 and orange coloured is EB27.

**Figure 2:**
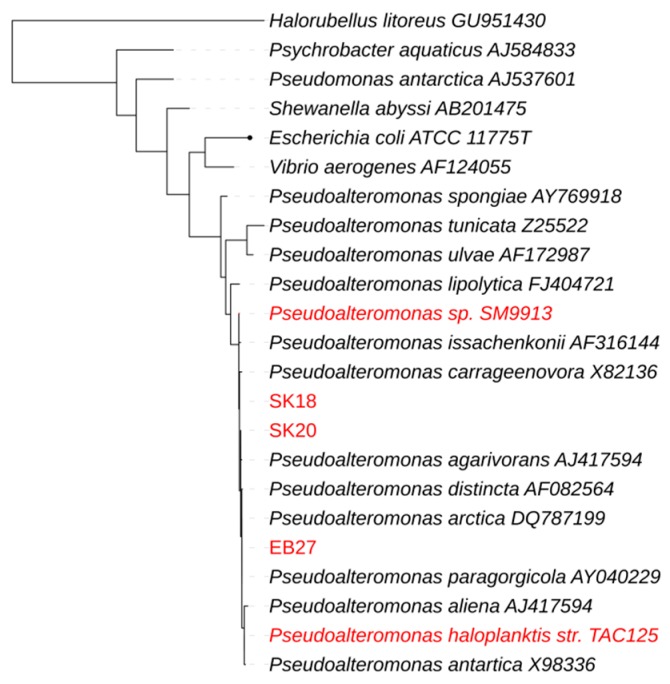
Phylogenetic comparison of the isolates investigated and reference strains (marked in red are the isolates that are used for this study. Maximum likelihood bootstrap consensus tree from 1000 replicates, calculated with MEGA6.0 [53] and visualized with iTOL [54,55]).

The Bacterial Pan Genome Analysis (BPGA) pan-genome pipeline was used to compare the whole genome sequences (Figure 1) [49]. *Pseudoalteromonas haloplanktis* TAC125 was used as a reference strain representing a shallow water isolate and *Pseudoalteromonas* sp. SM9913 was used as a deep sea reference strain as it had been retrieved from a deep sea sediment sample (1855 m) [21,46]. A phylogenetic comparison of the 16S rRNA gene of the isolates, reference strains, and a number of relevant type strains defined our isolates as true *Pseudoalteromonas* spp. (Figure 2). The 16S rRNA genes from the reference strains used for the whole genome comparison in this study were identified from the respective genomes by RNAmmer [50]. Due to the not fully assembled genomes (only one 16S rRNA copy in SK18 and EB27) of the isolates SK18, SK20, and EB27, the 16S rRNA gene sequences were retrieved by PCR. A pan-genome analysis, based on the comparison of all translated protein sequences, was then performed. The number of translated protein sequences present ranged from 3422 for *Pseudoalteromonas haloplanktis* TAC125 to 3941 for *Pseudoalteromonas* sp. EB27, with 2482 of these proteins being orthologs; making up 72.5% of the smallest genome and 62.9% of the largest genome. The number of unique proteins or paralogs is quite large, ranging from 308 to 809. The sponge isolates share 10 protein clusters not found in the free-living reference strains TAC125 and SM9913. These clusters include genes potentially encoding cation efflux proteins, integrases, recombinases, and proteins potentially involved in multidrug resistance and which may play a role in helping the *Pseudoalteromonas* strains adapt to life inside the sponge, and in helping them cope with other microorganisms inhabiting the sponge. For example, recombinases and integrases are known to mediate horizontal gene transfer, which is believed to play a key role in the genomic evolution of symbionts [51]. When comparing the individual isolates to the reference strains, EB27 shared 183 clusters with TAC125 and only 28 with SM9913, while SK18 shared 14 clusters with TAC125 and 75 clusters with SM9913. In addition, SK20 shared 60 clusters with TAC125 and 120 clusters with SM9913.

**Figure 3:**
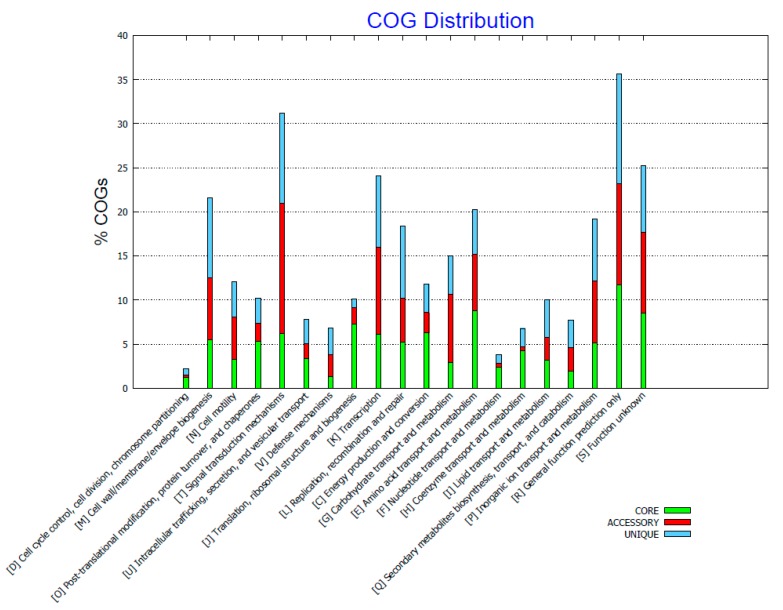
Cluster of orthologous groups (COG) distribution of the core, accessory, and unique genes of the five investigated *Pseudoalteromonas* genomes (generated with [49]).

The distribution of the protein clusters of orthologous groups (COG) affiliated with biological functions can be seen in Figure 3 (generated with [49]). The unique genes as mentioned earlier make up in total approximately 8.5 to 20% per genome. The potential function of these genes appears to be widespread and affiliated with many different cellular functions such as signal transduction mechanisms, cell wall, membrane and envelope biogenesis, recombination and repair, and many with only general or unknown function; so that no obvious pattern is evident. The accessory genes appear to be affiliated with several functions such as signal transduction mechanisms and defense mechanisms, and proteins with either an as yet unknown function or only a general prediction, but with again no obvious links with a specific function. The core genome mainly contributes towards cell cycle control, cell division, chromosome partitioning, translation, ribosomal structure, biogenesis, and nucleotide transport and metabolism (Figure 3). According to the COG distribution the Kyoto Encyclopedia of Genes and Genomes (KEGG) distribution of the translated genomes can be found in the Supplementary Figure S2.

**Figure 4:**
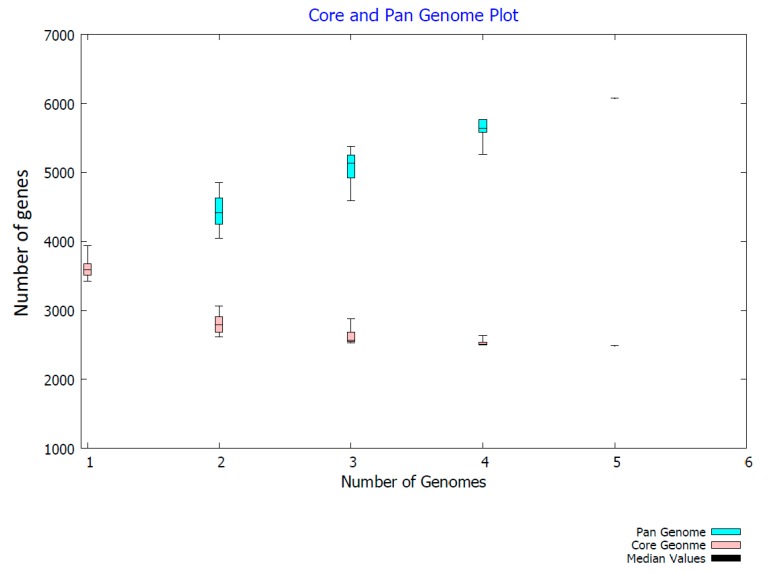
Core vs. pan-genome size plot generated with Bacterial Pan Genome Analysis BPGA tool [49].

The pan-genome analysis revealed an open pan-genome for the five *Pseudoalteromonas* isolates investigated here. Therefore, the number of dispensable or accessory genes is orders of magnitude larger than the size of the core genome and increases with the number of additional genomes (Figure 4), as defined by [56]. For the five genomes, the pan genome contains 6077 genes and the core genome is made up of 2482 genes. This is in line with recent findings for other non-pigmented *Pseudoalteromonas* spp. [43].

The genome sequences were then manually screened for genes encoding enzymes of potential industrial interest and were found to be quite rich in potential lipases/esterases and proteases, and to contain a relatively small number of potential β-galactosidase, β-glucosidase, and cellulase genes (Table 4). The number of potential genes encoding these enzyme activities does not, however, reflect the phenotypes seen in the plate screening assays (Table 1). For example, the presence of β-glucosidase genes does not necessarily lead to a positive phenotypic assay for this enzyme activity; all investigated *Pseudoalteromonas* strains contain at least two β-glucosidase genes, except for TAC125 which hosts none, but only EB27 displays this enzyme active in the plate screenings. However, the increased number of potential β-glucosidase- and cellulase-encoding genes in the genome of EB27 may account for the positive screening results in the plate assays for these enzyme activities.

**Figure 5:**
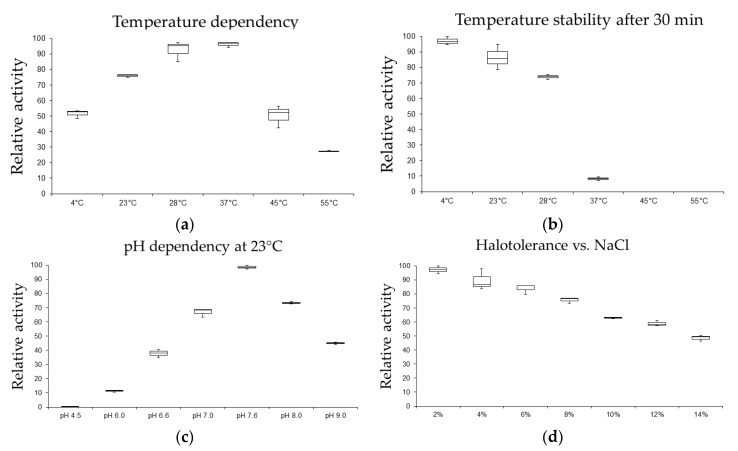
Optimal temperature (**a**), temperature stability (**b**), pH dependency (**c**) and halotolerance (**d**) assay results of a heterologously expressed β-glucosidase from the *Pseudoalteromonas* sp. EB27. The highest activity per assay was set as 100% activity and the other values were calculated accordingly. The highest total activity was measured as 2.5 U/mg of β-glucosidase at pH 7.6 and 28 °C.

EB27 contained two genes encoding periplasmic β-glucosidases and two genes which were annotated solely as β-glucosidases. The two β-glucosidase annotated genes were similar in size (1329 bp, 443 aa) and shared 72% nucleotide and 71% protein identity. Based on the strong β-glucosidase activity observed in EB27, one of the two β-glucosidase genes was cloned using PCR and subsequently subcloned into the *Escherichia coli* heterologous expression system. Following purification of the recombinant β-glucosidase, initial biochemical characterisation experiments investigating optimal temperature, temperature stability, pH dependency, and halotolerance were conducted to assess its potential for industrial applications (Figure 5). The heterologously expressed β-glucosidase from EB27 was most active at pH 7.6 with optimal temperatures ranging from 28 °C to 37 °C. Good activity was also observed at 4 °C, 23 °C, and 45 °C. The enzyme was found to be quite thermolabile, losing 90% of its activity following incubation at 37 °C for 30 min (Figure 5). The optimal temperature profile and the observed thermostability of the β-glucosidase would indicate that the enzyme is psychrophilic, being active at low temperatures, but readily inactivated at slightly elevated temperatures (28 °C and above). The halotolerance of the enzyme was assessed at various salt concentrations ranging from 2 to 14% sodium chloride. The enzyme loses approximately 25% of its activity in the 2 to 8% salt concentration range, with further decreases in activity being observed at higher salt concentrations (Figure 5). The recombinant enzyme would, therefore, appear to be halotolerant rather than halophilic.

Deferred antagonism-based antimicrobial assays were also performed in an effort to determine whether the three *Pseudoalteromonas* strains displayed any bioactivity against clinically-relevant test strains. The isolates were grown on both low and rich nutrient media and then overlaid with a number of clinically relevant test strains such as *Escherichia coli* 12210, *Staphylococcus aureus* NCD0 949, *Bacillus subtilis* 1E32, *Pseudomonas aeruginosa* PA-O1, *Acinetobacter johnsonii* WH00185, *Enterobacter faecium* NCIMB 11508, *Klebsiella pneumonia* NCIMB 13218, and *Enterobacter aerogenes* NCIMB 10102 in soft LB-agar. No bioactivity was observed, despite the fact that all three *Pseudoalteromonas* genomes contained at least one potential bacteriocin gene cluster (EB27 contained two bacteriocin gene clusters), with SK18 and EB27 also containing potential arylpolyene and siderophore encoding gene cluster (Table 5).

**Figure 6:**
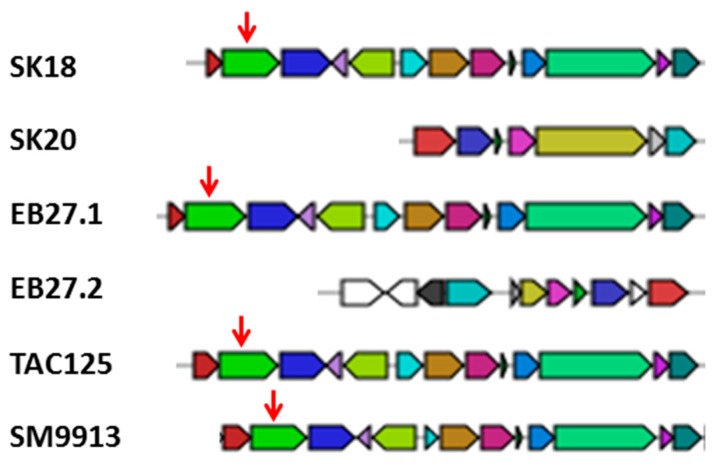
Organisation of the Bacteriocin gene clusters found in the investigated genomes (adapted from antiSMASH [57,58,59]). Red coloured genes are biosynthetic genes, blue coloured transport-related genes, green coloured regulatory genes and grey coloured other/unidentified genes. The red arrow points add the gene containing a tetratricopeptide repeat.

In addition, when TAC125 and SM9913 were subsequently analysed, one potential bacteriocin gene cluster was found to be present and highly conserved between the different isolates and the reference genomes (Figure 6). This gene cluster consists of 13 different genes with an average total size of 10.8 kb, except in SK20, in which the cluster only consists of seven genes with a total of 6.1 kb (Figure 6). In addition to the conserved bacteriocin gene cluster that can be found in all isolates, EB27 has a second small bacteriocin gene cluster spanning 10 kb, which is considerably different than the other clusters and is not a reduced form of the conserved cluster as in SK20.

Given that these *Pseudoalteromonas* spp. had been isolated from different sources, such as sponges (EB27, SK18, SK20), deep sea sediment (SM9913), as well as from open Antarctic seawater, we decided to investigate the presence of potential eukaryotic-like proteins such as ankyrin-repeats (ANK) and tetratricopeptide repeats domain-encoding proteins (TRP) which are known to be enriched in symbiotic sponge-associated microorganisms [60] (Table 6). The genomes of all isolates were found to contain a small number of genes with ankyrin and tetratricopeptide repeats, which are present at much lower levels than might be expected from a true sponge symbiont, such as *Poribacteria* sp. which contain at least 23 genes with tetratricopeptides repeats in its genome [61].

## 3. Discussion

*Pseudoalteromonas* spp. are known to be multitalented with respect to the production of enzymes of industrial interest; for example agarases [9], galactosidases [6], proteases [62], subtilases [10], and phospholipases [8] have been described from this genus. Furthermore some isolates are also able to produce acidic exopolysaccharides involved in biofilm formation [63], as well as antimicrobial compounds [64,65]. In general, the *Pseudoalteromonas* genus can be divided into pigmented and non-pigmented species, with the former producing mostly antimicrobial and antifouling compounds, and the latter being more versatile in the production of different enzymes [3]. The *Pseudoalteromonas* spp. isolates described herein are naturally non-pigmented and are, therefore, no exception to the aforementioned general classification as they produce a variety of different enzymes, but display no antimicrobial activity under the assay conditions tested, but interestingly they do possess potential bacteriocin and siderophore gene clusters in their genomes (Table 5, Figure 6).

Having isolated a number of *Pseudoalteromonas* strains from deep sea sponges, we decided to employ a number of approaches including plate screening, whole genome sequencing, and comparative genomics in an attempt to identify genes encoding enzymes with potentially biotechnologically relevant properties. The isolates were found to be cold adapted rather than true psychrophiles, according to the observed growth rates and doubling times at various temperatures (Table 2), and expressed a number of different enzyme activities, including β-glucosidase, β-galactosidase, protease, and lipase activities (Table 1). The isolates grew best at 23 °C and 28 °C with doubling times ranging from 20 to 40 min, compared to a generation time of 20 min at 37 °C for the mesophilic *E. coli* K-12 strain MG1655 [66], which shows that our isolates are capable of achieving similar growth rates to *E. coli* at these temperatures, supporting the feasibility of using *Pseudoalteromonas* as an expression system for cold adapted enzymes as previously demonstrated by Papa and colleagues [67]. 

Good activity levels for the recombinant β-glucosidase enzyme were observed at temperatures ranging from 4 °C to 37 °C, with optimal activity at 28 °C to 37 °C at a pH of 7.6 (Figure 5). Furthermore, the β-glucosidase was quite thermolabile, losing 30% of its activity after 30 min of incubation at 28 °C and 90% of its activity after 30 min of incubation at 37 °C. While β-glucosidases are typically involved in important processes in bacteria, such as degradation of cellulose and other carbohydrates for nutrient uptake, there is an increased interest in their use in the conversion of lignocellulosic biomass into reducing sugars for ethanol production. While at least two other types of enzymes are also required for the complete degradation of cellulose, namely the endoglucanases and cellobiohydrolases; β-glucosidases are believed to be the rate-limiting enzyme in these processes [68]. They also find industrial applications in wine making where they play a key role in the enzyme-mediated release of aromatic compounds from glycosidic precursors present in fruit juices, musts, and fermenting products. They are also used in flavour enhancement to improve the organoleptic properties of citrus fruit juices to reduce bitterness [69]. While the majority of β-glucosidases currently in use are mostly fungal in origin, bacterial derived enzymes are receiving increased recent interest, particularly for biofuel production applications [69]. Furthermore, enzymes from *Pseudoalteromonas* have proven useful in the hydrolysis of carbohydrates from algal biomass under alkaline conditions, which is uncommon for terrestrial β-glucosidases and could be used for biofuel production from marine sources [69,70,71].

As mentioned earlier, members of the genus *Pseudoalteromonas* are routinely isolated from a variety of different marcoorganisms. While they have also been isolated from sea water and sediment, they are usually found in association with macroorganisms [3,72]. With this in mind, we investigated the genomes of our three deep sea sponge associated *Pseudoalteromonas* strains, together with two free-living isolates for the presence of potential symbiosis genes, such as genes mediating microbe–host interactions (genes containing eukaryotic-like domains, like ankyrin and tetratricopeptide repeats) or those that may be beneficial in the acquisition or production of nutrients such as proteases, sulfatases or peptidases for the host or the symbiont [61,73]. While the genomes of the sponge-associated and free-living *Pseudoalteromonas* sp. isolates were rich in proteases and sulfatases, they lacked large numbers of genes encoding potential ankyrin and tetratricopeptide repeats (Table 6). Interestingly one of the genes which did contain a tetratricopeptide repeat is part of the conserved bacteriocin gene cluster that is found in all isolates (Figure 6), which provides some limited evidence of potential microbe–host interactions. In this respect, it is known that some bacteriocins are involved in mediating microbe–host interactions via biofilm formation on which a host can settle [74]. However, the lack of appreciable numbers of genes containing eukaryotic-like domains amongst the genomes of the of *Pseudoalteromonas* sponge isolates appears to suggest that they may not in fact form a true symbiotic relationship with their host. These *Pseudoalteromonas* isolates may, however, be indirectly beneficial to the sponge by breaking down polysaccharides or other nutrient containing materials and thereby making these available to both themselves and to the sponge; by functioning as either a commensal or transiently associated microbe. 

The pan-genome of the investigated isolates is open and each genome contains 8.5 to 20% unique genes and the core genome comprises of 2482 genes (Figure 1), whereas the whole pan genome comprises of 6077 genes. The possible functions of the translated genes have been investigated by assigning them to clusters of orthologous function. The distribution of the unique, accessory, and core genes is widespread across different biological functions and no obvious pattern is evident, besides core functions that seem to be conserved in all genomes such as cell cycle control, cell division, chromosome partitioning, translation, ribosomal structure, biogenesis, and nucleotide transport and metabolism (Figure 3). 

## 4. Conclusions

The following a comparative genomic analysis of non-pigmented sponge associated *Pseudoalteromonas* sp. isolated from different depths and free-living *Pseudoalteromonas* sp. we have demonstrated that these strains share a large open pan-genome and possess a considerable number of unique genes which is in line with results of other genome comparison of non-pigmented *Pseudoalteromonas* spp. [43]. We were unable to obtain definitive evidence based on these genome comparisons that non-pigmented *Pseudoalteromonas* spp. form true symbiotic relationships with deep sea sponges. While these non-pigmented strains do not appear to produce antimicrobial compounds, they do, however, produce a wide variety of different degradative enzymes, such as proteases, lipases, β-glucosidases, and β-galactosidases. A β-glucosidase gene was cloned from *Pseudoalteromonas* EB27 and heterologously expressed in *Escherichia coli.* Biochemical characterization of the recombinant β-glucosidase enzyme revealed enzyme characteristics with potentially useful biotechnological applications. For example, various food processing and bioremediation processes require halotolerant enzymes [75], while cold-active enzymes find uses in food production, detergents and in molecular biology [76]. There is also interest in the biotechnological application of β-glucosidases in relation to plant-based foods, such as their use in the conversion of phytoestrogen glucosides in fruits and vegetables to aglycone moieties and the removal of bitter compounds from citrus fruit juices [77]. The three *Pseudoalteromonas* sp. strains also possess good levels of extracellular lipase and protease activity; two important classes of industrial enzymes with projected global markets set to reach $590M USD and $2.21B USD by 2020 and 2021, respectively [78]. Alkaline proteases in particular are important industrial enzymes with application in detergents. Production of these enzymes is heavily dependent on the growth of the producer strain at pH of >7.5 [75]. The characteristics of the extracellular lipases, proteases, and β-glucosidase (cold-active, halotolerant, alkaline-active) produced by these *Pseudoalteromonas* strains together with their short doubling time at mesophilic temperatures, make these isolates an attractive source for further investigation. 

## 5. Materials and Methods

### 5.1. Sponge Collection and Isolation of Microorganisms

The sponges (*Poecillastra compressa*, *Inflatella pellicula* and *Sericolophus hawaiicus*) used for the isolation of microorganisms have been collected of the west coast of Ireland during the Biodiscovery cruises 2010 and 2013 by the remotely operated vehicle *Holland I* on board the R.V. *Celtic Explorer*. The sponges were rinsed directly after collection with sterile artificial sea water (3.33% (*w*/*v*) Instant Ocean, Aquarium Systems) to remove any exogenous material and were subsequently stored at −80 °C until further processing. The isolation of microorganisms was performed as follows; small sponge pieces were macerated with a sterile razor blade and serially diluted with artificial seawater and plated onto Starch-Yeast Extract-Peptone Seawater (SYP-SW) plates (10 g/L starch, 4 g/L yeast extract, 2 g/L peptone, 33.3 g/L artificial sea salt, 1.5% agar). The plates were inspected daily for colonies and incubated for four weeks at 28 °C. All colonies were restreaked until pure cultures were obtained.

### 5.2. Enzyme Activity Plate Screenings

The pure cultures were tested for different enzyme activities. All screenings were carried out at 28 °C and incubated for three to four days.

Protease screening was carried out using SYP-SW plates supplemented with 2% skim milk (Sigma-Aldrich, Arklow, Ireland), a clear halo around the colonies after incubation indicates a possible protease activity. Positive colonies were further tested on SYP-SW plates supplemented with 40 ng/mL X-Gal to differentiate between true protease activity and β-glucosidase/β-galactosidase activity, a blue colour change of the colony would indicate that it is rather the latter activity.

Cellulase activity was tested using SYP-SW plates supplemented with 0.1% Ostazin brilliant red hydroxyethyl-cellulose (OBR-HEC; Slovak Academy of Science, Institute of Chemistry); clear halos around colonies indicate cellulase activity.

Lipase activity was investigated via adding 1% tributyrin (Sigma-Aldrich) to the SYP-SW plates, again a clear halo around the colonies indicates a lipase or esterase activity.

### 5.3. Enzyme Assays and Growth Characterization

The β-glucosidase activity of the heterologously expressed enzyme was measured using 5 µg of protein, 20 µL 0.1 M p-nitrophenyl-β-d glucopyranoside as substrate and 1 mL of 0.1 M potassium phosphate buffer solution (pH 7.6). The obtained solution was incubated at 23 °C for 10 min and the absorbance at 420 nm was measured subsequently. The temperature and the pH of the potassium phosphate buffer were varied for the optimal temperature assays (tested were 4 °C, 23 °C, 28 °C, 37 °C, 45 °C, and 55 °C) and the pH dependency assay (tested were pH 4.5, 6.0, 6.6, 7.0, 7.6, 8.0, and 9.0). According to the Beer–Lambert law, the optimal activity of the β-glucosidase was approximately 2.5 U/mg, where one unit of β-glucosidase corresponds to the release of 1 µmol of p-nitrophenyl min^−1^ (ε = 1.6 × 10^4^ M^−1^ cm^−1^) in the reaction mixture under the assay conditions 28 °C and pH 7.6.

Overnight cultures incubated at 28 °C and 180 rpm were diluted the next day in 30 mL marine broth (Difco marine broth 2216) to an optical density at 600 nm of 0.05 and subsequently incubated at different temperatures (4 °C, 23 °C, 28 °C, 37 °C) in shaking incubators. The growth was monitored hourly by measuring the optical density at 600 nm. The specific growth rate (mu) and the generation/doubling time (t_gen_) was calculated using the formula mu = (ln(X_1_) − ln(X_0_))/(t_1_ − t_0_) and t_gen_ = (0.693/mu) × 60, with X_0_ being the optical density at the beginning of the exponential growth phase (approximate OD_600_ of 0.15), X_1_ a time point within the exponential growth phase (OD_600_ between 0.15 and 1.0) and t the time passed between X_0_ and X_1_ in hours.

### 5.4. Genomic DNA Isolation and Sequencing

Genomic DNA isolation was carried out by processing a 10 mL overnight culture (SYP-SW medium, 28 °C, 180 rpm), after centrifugation the media was removed and 2 mL lysis buffer (2% SDS, 1% CTAB, 100 mM Tris, 100 mM EDTA, 1.5 M NaCl, pH 8.0) were added and incubated in a water bath at 70 °C with occasional mixing for two hours. The cell lysate was centrifuged until a clear lysate was obtained, 0.7 volumes of Isopropanol were subsequently added to precipitate the genomic DNA (30 min, room temperature). After centrifugation, the supernatant was discarded and the obtained pellet was washed with 70% ethanol, then centrifuged again and after supernatant removal, air dried and finally resuspended in an appropriate amount of Tris-EDTA buffer (10 mM Tris, 1 mM EDTA, pH 8.0). DNA quality was assessed by running 5 µL DNA on an agarose gel resulting in a high molecular weight band. Purity of DNA was measured on a NanoDrop ND-1000 Spectrophotometer (Thermo Fisher Scientific, Waltham, MA, USA). DNA was quantified with a Qubit dsDNA HS assay (Thermo Fisher Scientific) prior to preparing genomic libraries with the Nextera XT DNA Library Prep Kit (Illumina, San Diego, CA, USA) according to the manufacturer’s instructions. Final libraries were barcoded with Nextera XT indices, assessed on a Bioanalyzer High Sensitivity DNA chip (Agilent Technologies, Santa Clara, CA, USA) and sequenced together on an Illumina MiSeq platform using paired-end 300 bp chemistry. Raw sequence data was quality trimmed using Trimmomatic version 0.36 [79], removing short reads and trimming both ends of reads containing low quality bases. Quality trimmed reads were assembled using SPAdes version 3.7.0 [80] in paired-end mode with default settings. The genome completeness was assessed using CheckM [81]. Full length 16S rRNA gene sequences were predicted for the reference strains using RNAmmer [50]. 16S rRNA gene sequences from the isolates SK18, SK20, and EB27 were obtained using universal 27f/1492r primer for PCR. (The genomes are deposited in the NCBI database under the accession numbers MTQB00000000, MTQC00000000, MTQD00000000).

### 5.5. Cloning, Expression, and Purification of the β-Glucosidase

The full length β-glucosidase was amplified using primers incorporating enzyme restriction sites and allowing in-frame cloning into the pBAD/mycHIS-A overexpression vector (Invitrogen, Carlsbad, CA, USA). Forward primer fBETA2 (TATATATC*ATG*ACAAAAATATCATTACCATTTAATTCACC) incorporates a *BspH*I restriction site (underlined) at the start codon (italics) of the gene. Reverse primer rBETA2 (ATATATAAGCTTTCTATTTGAGATAAGTGCTTTATAGG) incorporates a *Hind*III restriction site replacing the stop codon of the β-glucosidase gene. The amplified PCR product was digested with *BspH*I and *Hind*III, pBAD/mycHis-A was digested with *Nco*I and *Hind*III and subsequently an overnight ligation was carried out at 4 °C at a 10:1 ratio insert to plasmid. Two µL of the ligation reaction were added to 50 µL TOP10 chemically competent cells (ThermoScientific) and the transformation was carried out according to the manufacturer’s guidelines. The transformation mixture was plated in different amounts on LB plates containing 50 µg/mL ampicillin, 0.2% arabinose and 40 mg/mL X-GAL.

The β-glucosidase enzyme was purified from the overnight culture, supplemented with 100 µg/mL ampicillin and 0.02% arabinose, using the HisPur Ni-NTA Resin (Thermo Fisher Scientific). 50 mL of overnight culture was used and centrifuged at 4000× *g* for 15 min at 4 °C. The pellet was resuspended in 2 mL of Tris-EDTA buffer and 100 µL of lysozyme (10 mg/mL) was added and incubated at 37 °C for 15 min. After this, the obtained solution was subjected to three freeze-thaw cycles of 15 min each. After this, the lysate was centrifuged at 12,000× *g* for 30 min at 4 °C and the supernatant was collected. The HisPur Ni-NTA Resin (2 mL) was packed in a column and was equilibrated with 4 mL of equilibration buffer (20 mM sodium phosphate, 300 mM sodium chloride, 10 mM imidazole, pH 7.4). The protein lysate was mixed with an equal volume of equilibration buffer and was loaded twice onto the Nickel-Nitrilotriacetic acid Ni-NTA column. The column was washed subsequently three times with 4 mL of wash buffer (PBS, 25 mM imidazole, pH 7.4. The bound protein was eluted with twice with 4 mL of elution buffer (PBS, 250 mM imidazole, pH 7.4). The obtained protein elute was concentrated using VWR Vivaspin concentrator columns with a molecular weight cutoff of 10 kDa.

### 5.6. Genome Analysis and Comparison

The draft genomes were annotated using the RAST pipeline [82,83,84]. The genomes of *Pseudoalteromonas haloplanktis* TAC125 [46] and *Pseudoalteromonas* sp. SM9913 [21] were used as reference genomes for comparison and annotated again in the same manner as the newly isolated *Pseudoalteromonas* spp. to rule out annotation biases between different software packages. Genome comparison was carried out by using the BPGA pipeline [49] and manually screening the genomes for enzymes of industrial interest. The genomes were screened for secondary metabolite gene clusters using antiSMASH [57,58,59].

## Figures and Tables

**Table 1:** Enzyme active profile of the *Pseudoalteromonas* sp. isolates based on plate screenings. Activity is depicted as ‘X’ and intensity is indicated by the number of ‘Xs’, with ‘X’ low activity and ‘XXX’ describing highest activity. (Glc = β-glucosidase, Gal = β-galactosidase).

Isolate ID	Sponge	Depth (m)	Cellulase	Lipase	Protease	β-Glc/Gal
EB27	*Poecillastra compressa*	1480	XX	X	XXX	XXX (Glc)
SK18	*Sericolophus hawaiicus*	2129	-	X	XXX	-
SK20	*Inflatella pellicula*	2900	-	X	-	XXX (Gal)

**Table 2:** Growth characteristics (specific growth rate and generation time) of the *Pseudoalteromonas* sponge isolates at different temperatures (*n* = 3), including standard errors. Mu (specific growth rate) = (ln(X_1_) − ln(X_0_))/(t_1_ − t_0_).

ID	4 °C mu; t_gen_ (min)	23 °C mu; t_gen_ (min)	28 °C mu; t_gen_ (min)	37 °C mu; t_gen_ (min)
EB27	0.54 ± 0.15; 88.3 ± 20.01	1.03 ± 0.05; 40.6 ± 2.1	0.98 ± 0.04; 42.46 ± 1.84	0.82 ± 0.09; 51.39 ± 6.75
SK18	0.28 ± 0.06; 159.36 ± 12.75	1.76 ± 0.095; 23.8 ± 1.24	2.08 ± 0.013; 20 ± 0.13	1.46 ± 0.16; 29.04 ± 2.9
SK20	0.29 ± 0.02; 144.92 ± 31.2	0.99 ± 0.08; 42.36 ± 3.56	1.41 ± 0.01; 29.5 ± 0.24	0.9 ± 0.14; 48.66 ± 7.29

**Table 3:** Genome sequencing statistics and genome features of reference strains. (CDS, coding DNA sequences, N50 weighted median length of the sequences making up 50% of genome size).

ID	Genome Size (Mb)	GC Content	N50 (kb)	Contigs	CDS	No. of RNAs	Coverage	Genome Completness (%)
TAC125	3.85	40.1%	n/a	n/a	3473	134	n/a	100
SM9913	4.04	40.3%	n/a	n/a	3699	87	n/a	100
EB27	4.56	39.1%	216.9	114	4012	136	196×	99.72
SK18	3.98	40.2%	156.5	115	3582	110	213×	99.75
SK20	4.15	40.3%	98.5	213	3811	139	230×	99.66

**Table 4:** Abundance of genes encoding for enzymes of potential industrial interest.

ID	Lipase/Est.	β-Galactosidase	Protease	β-Glucosidase	Cellulase
TAC125	49	0	35	0	2
SM9913	67	0	42	2	3
EB27	69	1	48	4	5
SK18	63	0	39	2	3
SK20	56	1	40	2	3

**Table 5:** Abundance of secondary metabolite gene clusters.

ID	Bacteriocin	Arylpolyene	Siderophore
TAC125	1	1	-
SM9913	1	-	1
EB27	2	1	-
SK18	1	1	1
SK20	1	-	-

**Table 6:** Abundance of genes suggested being involved in a symbiotic relationship.

ID	Ankyrin Repeats	Tetratricopeptide Repeats	Nitrite Reductase	Proteases	Sulfatases	Peptidases
TAC125	2	2	1	35	0	58
SM9913	1	2	0	42	1	63
EB27	2	2	3	48	0	65
SK18	1	2	0	39	1	63
SK20	1	2	0	40	1	58

## Supplementary Materials

The Supplementary files are available online at www.mdpi.com/1660-3397/15/6/184/s1.
