# Supplementary material for: Biotechnological Potential of Cold Adapted Pseudoalteromonas spp. Isolated from ‘Deep Sea’ Sponges
Source: Mar Drugs. 2017 Jun 19;15(6):184. doi: 10.3390/md15060184 (PMC5484134; doi:10.3390/md15060184)
Supplement: Supplementary file 1 [file marinedrugs-15-00184-s001.pdf]

Biotechnological potential of cold adapted *Pseudoalteromonas* spp. isolated from 'deep sea' sponges

Erik Borchert[1], Stephen Knobloch[2], Emilie Dwyer[1], Sinéad Flynn[1], Stephen A. Jackson[1], Ragnar Jóhannsson[2], Viggó T. Marteinsson[2], Fergal O'Gara[1,3,4] and Alan D.W. Dobson[1]*

Supplementary figure 1

Semi-quantitative enzyme activity assays for protease and β-galactosidase activity

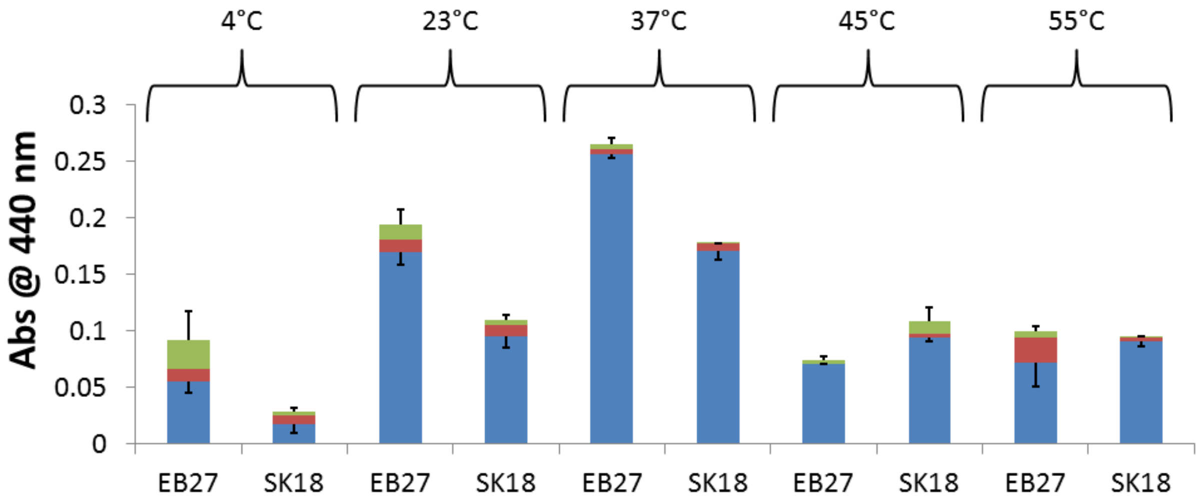

Figure 1A: Protease activity assay of the isolates SK18 and EB27 at different temperatures.

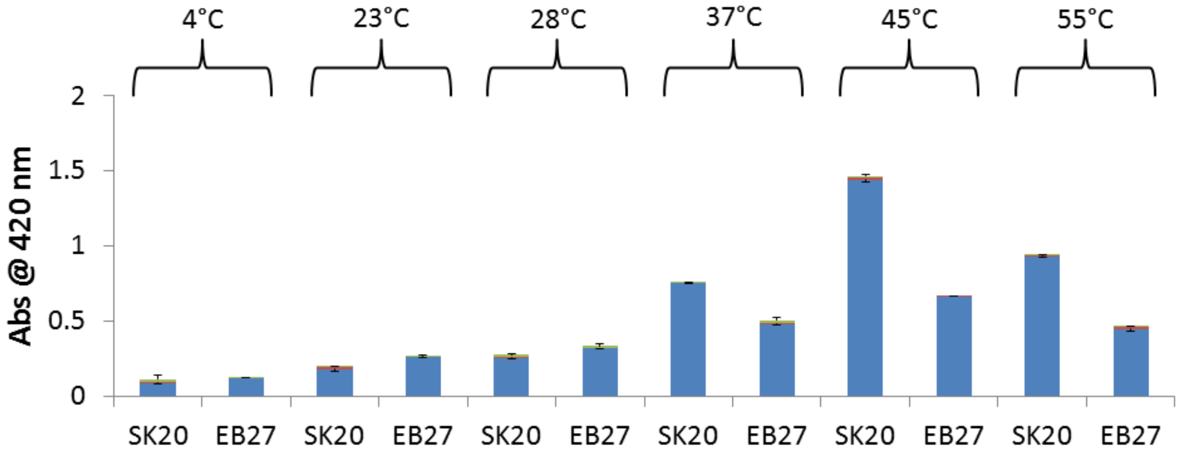

Figure 1B: β-galactosidase activity of the isolates SK20 and EB27 at different temperatures.

Materials and methods:

Native enzyme assays for β-galactosidase and protease activity were carried out with aliquots of overnight cultures from the respective *Pseudoalteromonas* sp. isolates. β-

galactosidase assays were carried out by adding 150 µl of overnight culture to 1350 µl 0.1 M potassium phosphate buffer solution (pH 8.5, pH 6.0 and 7.0 gave little to no detectable activity) and 22,5 µl 0.1 M p-nitrophenyl-β-d galactopyranoside as substrate, incubated for 1.5 h at different temperatures and absorbance at 420 nm was measured subsequently. For the protease assay 150 µl overnight culture were added to 250 µl 2% azocasein solution (0.1 M Tris-HCl, pH 8.0) followed by incubation for 1 h at different temperatures, then 900 µl 10% trichloroacetic acid were added and incubated for 15 min at room temperature to stop the reaction and afterwards centrifuged for 10 min at max. speed. 600 µl of the supernatant were combined with 700 µl 1 M NaOH and the absorbance was measured at 440 nm.

Supplementary figure 2

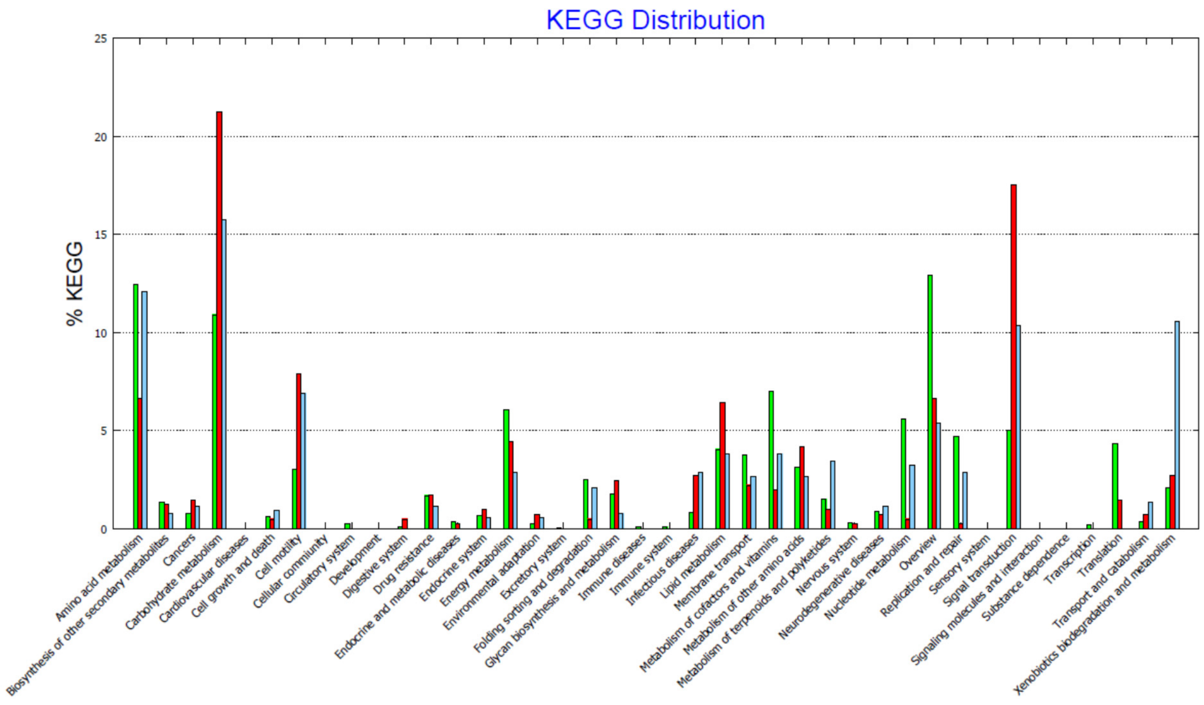

Figure S2. KEGG (Kyoto Encyclopedia of Genes and Genomes) functional distribution of the annotated genes. All genes from the five genomes are combined and separated into core, accessory and unique genes.
